# Leveraging Metaheuristic Unequal Clustering for Hotspot Elimination in Energy-Aware Wireless Sensor Networks

**DOI:** 10.3390/s23052636

**Published:** 2023-02-27

**Authors:** Hadeel Alsolai, Mashael Maashi, Muhammad Kashif Saeed, Abdullah Mohamed, Mohammed Assiri, Sitelbanat Abdelbagi, Suhanda Drar, Amgad Atta Abdelmageed

**Affiliations:** 1 Department of Information Systems, College of Computer and Information Sciences, Princess Nourah bint Abdulrahman University, P.O. Box 84428, Riyadh 11671, Saudi Arabia; 2 Department of Software Engineering, College of Computer and Information Science, King Saud University, P.O. Box 103786, Riyadh 11543, Saudi Arabia; 3 Department of Computer Science, Applied College, King Khalid University, Muhayil 63311, Saudi Arabia; 4 Research Centre, Future University in Egypt, New Cairo 11845, Egypt; 5 Department of Computer Science, College of Sciences and Humanities-Aflaj, Prince Sattam bin Abdulaziz University, Al-Aflaj 16733, Saudi Arabia; 6 Department of Computer and Self Development, Preparatory Year Deanship, Prince Sattam bin Abdulaziz University, Al-Kharj 16278, Saudi Arabia

**Keywords:** energy dissipation, load balancing, wireless sensor network, hotspot problem, unequal clustering, tuna swarm algorithm

## Abstract

Wireless sensor networks (WSNs) are becoming a significant technology for ubiquitous living and continue to be involved in active research because of their varied applications. Energy awareness will be a critical design problem in WSNs. Clustering is a widespread energy-efficient method and grants several benefits such as scalability, energy efficiency, less delay, and lifetime, but it results in hotspot issues. To solve this, unequal clustering (UC) has been presented. In UC, the size of the cluster differs with the distance to the base station (BS). This paper devises an improved tuna-swarm-algorithm-based unequal clustering for hotspot elimination (ITSA-UCHSE) technique in an energy-aware WSN. The ITSA-UCHSE technique intends to resolve the hotspot problem and uneven energy dissipation in the WSN. In this study, the ITSA is derived from the use of a tent chaotic map with the traditional TSA. In addition, the ITSA-UCHSE technique computes a fitness value based on energy and distance metrics. Moreover, the cluster size determination via the ITSA-UCHSE technique helps to address the hotspot issue. To demonstrate the enhanced performance of the ITSA-UCHSE approach, a series of simulation analyses were conducted. The simulation values stated that the ITSA-UCHSE algorithm has reached improved results over other models.

## 1. Introduction

Wireless sensor networks (WSNs) have developed into one of the auspicious technologies utilized in the modern era [1]. WSNs monitor the environments in which they are placed to gather data and can identify changes in monitoring sound, temperature, vibration and motion intensity, pressure, humidity, etc. [2,3]. WSN applications are commonly utilized in the smart home monitoring system, environmental observing systems, natural disaster monitoring systems, habitat monitoring systems, traffic monitoring systems, bridges or building operational monitoring systems, military solicitations, inventory management systems, bio-medical applications, health monitoring systems and industrial robotics systems [4]. WSNs can be either dynamic or static sensor nodes (SNs) or a combination of both. Certainly, SNs are energy-limited since they depend on batteries for energy sources. Owing to energy limitations, the lifespan of a WSN was also limited [5,6]. Due to the nature of applications where WSNs were leveraged, it was generally very tough to reach all nodes and replace their sensor battery. Hence, numerous protocols and techniques are being modeled to minimalize power utilization in all sensor nodes and to extend the network lifespan [7]. Numerous hole issues are detected in the WSN such as sink holes, energy holes, jamming holes, coverage holes and routing holes. The energy hole problems have a huge impact on the sensor network at the time of data transmission from source to destination [8,9].

To prevent the network from experiencing hotspot problems, unequal clustering (UC) methods are used for load balancing among cluster heads (CHs) [10]. UC diminishes the cluster size which is nearer to the base station (BS) and the cluster size rises since the distance between CH and BS increases [11]. Metaheuristic and classical methods were two broad areas of clustering techniques. Traditional clustering is split into five types: density-based, area-based, model-based, hierarchical and grid-based [12]. One difficulty of such techniques was to get stuck in local optimum effortlessly. Previously, several metaheuristic algorithms were leveraged to solve this weakness. Metaheuristic approaches can offer near-optimum solutions in less time compared to traditional techniques. Metaheuristic optimizers were found to be potential ways of solving complicated optimization issues [8]. The flexibility of metaheuristics illustrates the usage of such techniques in several ways without making any particular changes to the algorithm structure. As problems are black boxes in the metaheuristic technique, they are simply implemented to different problems [13]. In other words, only the outputs and inputs of a system were significant in a metaheuristic approach; therefore the only significant problem for the designer was how to implement the solution to the approach [14]. Actually, in metaheuristic techniques, optimization is performed by utilizing a set of solutions (population).

This paper devises an improved tuna-swarm-algorithm-based unequal clustering for hotspot elimination (ITSA-UCHSE) technique in an energy-aware WSN. The ITSA-UCHSE technique intends to resolve the hotspot problem and uneven energy dissipation in the WSN. In this study, the ITSA is derived from the use of a tent chaotic map via the traditional TSA. In addition, the ITSA-UCHSE technique computes a fitness value based on energy and distance metrics. Moreover, the cluster size determination via the ITSA-UCHSE technique helps to address the hotspot issue. To exhibit the enhanced performance of the ITSA-UCHSE algorithm, a series of simulation analyses were conducted. 

## 2. Literature Review

In [15], a hybrid-optimization-based unequal clustering with mobile sink (HOUCMS) technique was modeled as HOUCMS incorporated mobile sink, hybrid optimized methods, and UC. Firstly, for selecting CH, a method called butterfly optimization was utilized after the nodes were allocated to CHs depending on the competition radius principle in the UC approach. Furthermore, the route to sink could be determined by the ACO and, at last, the mobile sink utilized in HOUCMS collected the collected dataset from every CH. Agrawal et al. [16] designed an unequal clustering method that chooses probationary CH using FL, and the optimization of probationary CHs is achieved by leveraging the harmony search algorithm (HSA). The devised technique displays the dynamic ability of high search efficiency and the FL of HSA that expands the network lifetime.

Maheswari and Karthika [17] formulated a new secure UC protocol with IDS for attaining QoS parameters such as security, energy and lifetime. First of all, the devised method utilizes an adaptive neuro-fuzzy-related clustering method for choosing tentative CHs (TCH) utilizing three input variables such as distance to neighbors, residual energy and distance to BS. After, TCHs strive for the last CH and the best CH is chosen to utilize the deer hunting optimization (DHO) technique. Revanesh et al. [18] introduced a technique called directed acyclic graph-related trust aware load-balanced routing (DAG-BTLBR). At first, the SNs were clustered unequally in an energy-efficient way to minimalize hotspot issues through the emperor penguin colony (EPC) approach. After clustering, the packet is transferred using load-balanced routes, securely utilizing the adaptive neuro-based dual fuzzy (ANDual Fuzzy) mechanism that minimizes the power utilization by choosing the best secure routes.

Arjunan and Sujatha [19] presented a method called FL-related UC and ACO-oriented routing, a hybrid protocol for the WSN for extending the network lifetime and eliminating hotspot issues. This protocol has cluster maintenance, CH selection and inter-cluster routing. FL chooses CHs effectually and splits the network into UC depending on node degree, RE, node centrality and distance to BS. In [20], a hybrid of the FL technique and HAS were designed to extend the lifespan of the network. UC was a method projected by the researchers for managing the hotspot problem; later, it was added. The devised technique forms an unequal cluster. To prove the efficacy of the presented technique, certain renowned UC methods and harmony search-based techniques were utilized for comparing the presented technique under distinct network settings.

Ref. [21] presents a Bayesian sequential sensor placement method, which depends on the robust information entropy for multi-type sensors. This technique has two salient features. In [22], a novel cluster-tree routing technique for gathering data (CTRS-DG) was devised that has two layers: one is aggregation and reconstruction and another one is routing. A dynamic and self-organizing entropy-oriented clustering technique for selecting CH and cluster formation is presented in the aggregation and reconstruction layer. Wu et al. [23] devised a new technique named the event boundary detection algorithm (EEBD) which is based on lightweight entropy in the WSN. The EEBD can be independently executed on all wireless sensors for determining whether it is a boundary sensor node, by making a comparison of the entropy values against the threshold which relies upon the boundary width.

Anuradha et al. [24] introduced a new seagull optimization (SGO)-based unequal clustering (SGOBUC) method for accomplishing energy efficiency in WSNs. The SGOBUC method has derived a good fitness including various parameters so that energy efficiency can be executed. Sivakumar et al. [25] developed a technique named EAOGSO-UCP (energy-aware oppositional group search-optimizer-based unequal clustering protocol) for WSNs. The core objective of the EAOGSO-UCP method is to organize the network into a set of unequal clusters by making a proper selection of the unequal cluster sizes and CHs. Muthukkumar et al. [26] proposed a GA-based energy-aware multihop clustering (GA-EMC) method for heterogeneous WSNs (HWSNs). In HWSNs, each node has different initial energy and commonly has an energy consumption limitation. A GA determined the optimum CHs and their locations in the network. Chauhan and Soni [27] projected an EAUCA method (energy-aware unequal clustering algorithm) to enhance the lifetime of a network and diminish the energy holes. This EAUCA constitutes unequal-sized clusters so that clusters in the base station vicinity are smaller than the farthest. Though several unequal clustering techniques are available in the literature, the energy-efficient performance still needs to improve.

## 3. The Proposed Model

**Figure 1:**
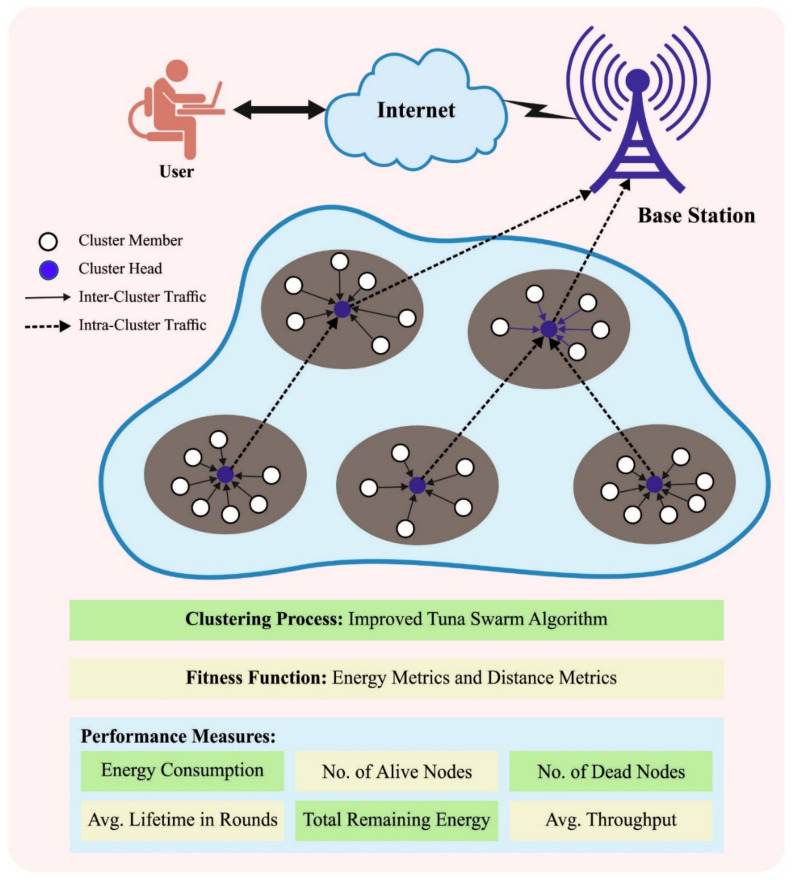
Overall procedure of ITSA-UCHSE system.

This paper has developed a novel ITSA-UCHSE method for hotspot elimination in energy-aware WSNs. The ITSA-UCHSE technique is intended to resolve hotspot problems and uneven energy dissipation in the WSN. Additionally, the ITSA is derived from the use of a tent chaotic map with the traditional TSA. In addition, the ITSA-UCHSE technique computes a fitness value based on energy and distance metrics. Figure 1 exhibits the overall procedure of the ITSA-UCHSE approach.

### 3.1. Energy Model

In this work, the WSN was adapted as follows: N node was installed at random in a square area whose side length was S. The node energy was limited and every node was immovable. A node might have changed the communication power. A receiver could notice the received signal intensity for estimating the distance to the sender. The energy consumption could be evaluated by using the following expression.

(1) $$E_{\mathrm{Tx}}\left(l,d\right)=\left\{\begin{array}{c} 1\times E_{\mathrm{elec}}+1\times \epsilon_{\mathrm{ts}}d^{2},\;d<d_{\mathrm{corssover}}\\ 1\times E_{\mathrm{elec}}+1\times \epsilon_{\mathrm{ts}}d^{2},\;d\geq d_{\mathrm{corssover}} \end{array}\right.$$

In Equation (1), l represents data whose unit was a bit and indicates the transmission distance. $E_{\mathrm{Elec}}$, $\varepsilon_{\mathrm{fs}},$ $\varepsilon_{\mathrm{mp}}$ and $d_{\mathrm{corssover}}$ denote the constants.

### 3.2. Design of ITSA

The TSA can be established as a new metaheuristic optimized system. Generating the place upgrade approach which makes procedure optimization searching feasible, simulations can be conducted of the foraging performance of tuna schools [28]. During this approach, the tuna schools pinpoint the food location that connects the global optimum solution with problems. Let N be individuals from the tuna school; the mathematical process utilized for replicating the foraging performance of schools of tuna is as follows:

(2) $$X_{i}^{t+1}=\left\{\begin{array}{c} \omega_{1}\cdot \left(X_{\mathrm{rand}\;}^{t}+\beta \cdot \left|X_{\mathrm{rand}\;}^{t}-x_{i}^{t}\right|\right)+\omega_{2}\cdot X_{i}^{t},i=1,\;\mathrm{rand}\;<\frac{t}{t_{\max}}\\ \omega_{1}\cdot \left(X_{\mathrm{rand}\;}^{t}+\beta \cdot \left|X_{\mathrm{rand}\;}^{t}-x_{i}^{t}\right|\right)+\omega_{2}\cdot X_{i-1}^{t},i=2,3,\ldots ,N,\;\mathrm{rand}\;<\frac{t}{t_{\max}}\\ \omega_{1}\cdot \left(X_{\mathrm{besi}\;}^{t}+\beta \cdot \left|X_{\mathrm{besi}\;}^{t}-X_{i}^{t}\right|\right)+\omega_{2}\cdot X_{i}^{t},i=1,\;\mathrm{rand}\;\geq \frac{t}{t_{\max}}\\ \omega_{1}\cdot \left(X_{\mathrm{besi}\;}^{t}+\beta \cdot \left|X_{\mathrm{besi}\;}^{t}-x_{i}^{t}\right|\right)+\omega_{2}\cdot X_{i-1}^{t},i=2,3,\ldots ,N,\;\mathrm{rand}\;\geq \frac{t}{t_{\max}} \end{array}\right.$$

(3) $$\omega_{1}=a+\left(1-a\right)\cdot \frac{t}{t_{\max}},$$

(4) $$\omega_{2}=\left(1-a\right)-\left(1-a\right)\cdot \frac{t}{t_{\max}},$$

(5) $$\beta =e^{\mathrm{bl}}\cdot \;\cos\;\left(2\pi b\right),$$

(6) $$l=e^{3\;\cos\;\left(\left(\left(t_{\max}+\frac{1}{t}\right)-1\right)\pi \right)},$$

where $X_{i}^{t+1}$ stands for the position of the $i_{\mathrm{th}}$ individual at t+1 iteration, t denotes the present iteration count, $\omega_{1}$ and $\omega_{2}$ imply the weighted parameters which lead the individual to move near the position of the optimum individuals and prior individuals, $X_{\mathrm{rand}}^{t}$ signifies the position of arbitrarily sampled individuals in the population and implies the arbitrary vector with [0–1] values, $X_{\mathrm{best}}^{t}$ signifies the position of the optimum individual (food) at t iterations, $t_{\max}$ stands for the maximal count of iterations, a signifies the constant which closely controls the individual that follows the optimum food and prior food from the primary stage, and b indicates the uniformly distributed arbitrary number betwixt zero and one.

Besides the spiral-shaped position upgrade process, the tuna also ensures a parabolic-type position upgrade employing the food as a reference point for improving this technique’s global search abilities. Considering that these two approaches are conducted concurrently, the selective probability is fixed at 0.5. The mathematical process was defined as follows.

(7) $$X_{i}^{t+1}=\left\{\begin{array}{l} X_{\mathrm{best}}^{t}+\;\mathrm{rand}\cdot \;\left(X_{\mathrm{besi}}^{t}-X_{i}^{t}\right)+\mathrm{TF}\cdot p^{2}\cdot \left(X_{\mathrm{besi}}^{t}-X_{i}^{t}\right),\;\mathrm{rand}<0.5\\ \mathrm{TF}\cdot p^{2}\cdot X_{i}^{t},\;\mathrm{rand}\geq 0.5\;’ \end{array}\right.$$

Chaos is a mathematical approach which is often utilized for enhancing exploration as well as improvement. In 2017, Suresh and Lal integrated a logistic chaotic map using Darwin PSO (DPSO) for producing a robust, dependable and quick approach to segment satellite images while continuing to improve the quality of pictures. Kohli and Arora related several variations in GWO and selective Chebyshev chaotic mapping to adapt the crucial parameter of GWO. The chaotic GWO considerably enhanced the reliability of global optimality and outcome quality. In 2020, simulated by the individual intelligence and sexual stimulation of chimpanzee group hunting, Khishe and Mosavi projected a chimp-optimized algorithm (Choa), whereas the semi-deterministic features of the chaotic map, respectively, boosted the improved ability of the Choa.

By initializing the TSA, one can primarily contain and arbitrarily create the location of tuna individuals in upper as well as lower bounds. This initialization can result in an unequal distribution of tuna throughout space, causing this technique for maturing to create a locally optimum solution prematurely. During this case, an initialized procedure dependent upon the tent chaotic sequence was provided as a solution to this problem. The segmentation linear mapping creates a tent chaotic sequence. Tent mapping with uniform distribution purpose and higher correlation allows this technique to readily escape in the optimum local solution, preserve the variation in populations and improve their capacity for global searching. Employing the sequence mapped in the range of zero and one, dependent upon the chaotic map, the tuna population was then initialized based on the chaotic feature. The mathematical equation of tent mapping is as follows:

(8) $$x_{n+1}=\left\{\begin{array}{c} \frac{x_{n}}{\alpha },0\leq x_{n}<\alpha \\ \frac{1-x_{n}}{\left(1-\alpha \right)},\alpha <x_{n}<1 \end{array},\right.\;$$

whereas the value range α in this article is 0.5. This method of initializing via the tent map is

(9) $$x_{i}^{\mathrm{int}}=x_{\min}+\mathrm{Chaos}*\left(x_{\max}-x_{\min}\right),\;$$

in which $x_{\max}$ and $x_{\min}$ denote the lower as well as upper limits of values of the independent variable, correspondingly as explained in Algorithm 1.
**Algorithm 1: Pseudocode for ITSA**Initializing the random population of individuals $x_{i}$ Allocate free parameters a and z    While $\left(t<t_{\;\max\;}\right)$    Compute the fitness values of individuals    Upgrade $X_{\mathrm{best}}^{t}$    For (each individual) do        Update $\omega_{1},\omega_{2},p$ using Equations (3) and (4).        If (and <z) then            Update the position $X_{i}^{t+1}$
        Else if (r and ≥z) then            If (and <0.5) then                If $(t/t_{\;\max\;}\mathrm{and}<$r) then                    Update the position $X_{i}^{t+1}$
                Else if $(t/t_{\;\max\;}\mathrm{and}\geq$r) then                    Update the position $X_{i}^{t+1}$
            Else if (r and ≥0.5) then                Update the position $X_{i}^{t+1}$
            End for        t=t+1    End while Return the optimal individual $X_{\mathrm{best}}$ and the optimal fitness value $F\left(X_{\mathrm{best}}\right)$


### 3.3. Process Involved in Unequal Clustering

The ITSA-UCHSE technique computes a fitness value based on energy and distance metrics. Initially, the node sends primary energy at a particular signal intensity and the node receives this message and computes the distance to every node [29]. Additionally, BS receive this message, and later calculates and broadcasts $E_{\mathrm{ave}}\cdot E_{\mathrm{ave}}$ denotes the average RE of a living node. CH is designated by reject radius $\left(R_{j}\right)\;$and competition radius $\left(R_{c}\right)$.

The formulation $R_{c}$ is presented as follows

(10) $$R_{t}=\left(1-0.3\times \frac{d_{\max}-\;d\left(i,\;\mathrm{BS}\right)}{d_{\max\;}-{\;d}_{\mathrm{nin}}}\right)\times R_{\max}$$

where $R_{\max}$ denotes the maximal $R_{c}$ which is defined in advance. $d_{\max}$ and $d_{\min}$ show the maximal and minimal of $d\left(i,\;\mathrm{BS}\right)$. $R_{c}$ reflects the effect of $d\left(i,\;\mathrm{BS}\right)$ clustering. no$\mathrm{de}\left(i,\;\mathrm{BS}\right)$e is smaller; then, the inter-cluster transmission load becomes heavy.

(11) $$R_{j}=\alpha \times \beta \times R_{c}$$

Now, α and β are variables. α reflects the effect of RE. The node has a large RE and is small in α. $E_{i}$ shows the RE of i-th nodes.

(12) $$\alpha =\left\{\begin{array}{c} \max\left[\frac{1}{2},\left(1+\frac{E_{\mathrm{ave}}-E_{i}}{E_{\mathrm{ave}}}\right)\right],{\;E}_{i}\geq E_{\mathrm{ave}}\\ \min\left[\frac{3}{2},\;\left(1+\frac{E_{\mathrm{ave}}-E_{i}}{E_{\mathrm{ave}}}\right)\right],E_{i}<E_{\mathrm{ave}} \end{array}\right.$$

β reflects the effect of the amount of ANs. The node with additional ANs can be small in β.

(13) $$\beta =\left\{\begin{array}{c} \max\left[\frac{1}{2},\left(1+\frac{N_{\mathrm{ave}}-N_{i}}{E_{\mathrm{ave}}}\right)\right],{\;N}_{i}\geq N_{\mathrm{ave}}\\ \min\left[\frac{3}{2},\;\left(1+\frac{N_{\mathrm{ave}}-N_{i}}{N_{\mathrm{ave}}}\right)\right],N_{i}<N_{\mathrm{ave}} \end{array}\right.$$

$N_{\mathrm{ave}}$ indicates the average amount of nodes within the circle of which the radius is $R_{c}$ which is evaluated as follows. $N_{i}$ indicates the number of nodes within the circle whereby the center is i and the radius is $R_{c}d\left(i,\;\mathrm{RNs}\right)$ and represents the average distance from i to this node.

(14) $$N_{\mathrm{ave}}=\frac{\pi \times N\times R_{c}^{2}}{S^{2}}$$

$t_{i}$ reflects the effect of $d\left(i,\;\mathrm{RNs}\right)$. A node having a lesser $d\left(i,\;\mathrm{RNs}\right)$ is small in it.

(15) $$t_{i}=\frac{d\left(i,\cdot {\mathrm{RN}}_{s}\right)}{R_{s}}\times t_{0}$$

where ${\;t}_{0}$ denotes a time constant. The node that has the opportunity of being a CH is called a CH candidate (CHC). Initially, every node waits for $t_{i}$. While waiting, a node continually receives messages. If node i-th receives a cluster-built message from q-th  nodes, it estimates the distance to q. If the distance is less than the sum of ${q^{\prime }R}_{c}i^{\prime }R_{j},$ then i becomes non-CHC. After waiting, if i has remained as a CHC, then it turns out to be a CH and transmits the cluster-built messages with the $R_{c}$ of i. The smaller the${\;R}_{j}$ is, the greater the chance that the node becomes CH. k indicates the optimal amount of CHs. When k CH is selected or waiting hours are over $t_{0}$, then CH selection can be completed. A non-CH node chooses a neighboring CH to join its cluster.

## 4. Experimental Validation

The unequal clustering results of the ITSA-UCHSE methodology are tested under diverse nodes in this section.

**Figure 2:**
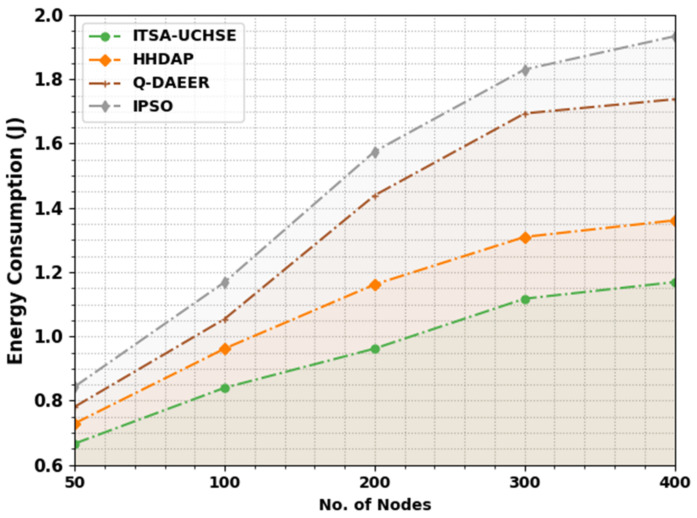
ECON analysis of ITSA-UCHSE system under distinct nodes.

Table 1 and Figure 2 exhibit the energy consumption (ECON) inspection of the ITSA-UCHSE technique with existing models [30]. The experimental outcome highlighted that the ITSA-UCHSE technique showed improved ECON values under every node. With 50 nodes, the ITSA-UCHSE technique obtained a reduced ECON value of 0.6666 J while the HHDAP, Q-DAEER and IPSO techniques reached increased ECON values of 0.7294 J, 0.7811 J and 0.8439 J, respectively. Additionally, with 400 nodes, the ITSA-UCHSE method gained a reduced ECON value of 1.1690 J while the HHDAP, Q-DAEER and IPSO approaches reached increased ECON values of 1.3611 J, 1.7379 J and 1.9337 J, correspondingly.

**Figure 3:**
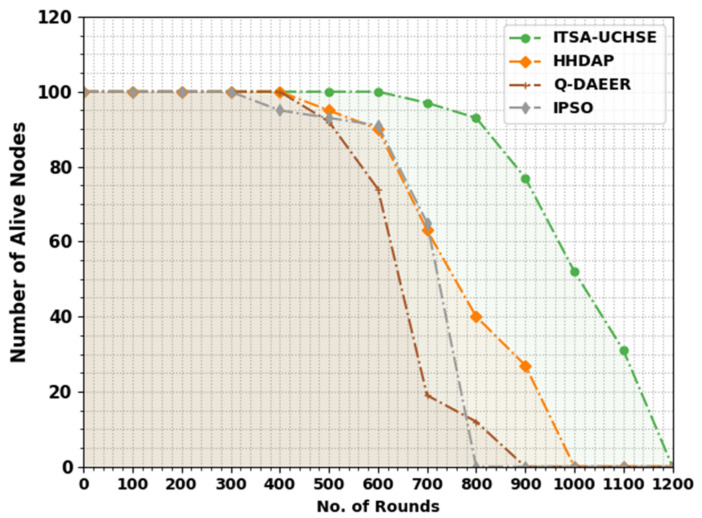
NOAN analysis of ITSA-UCHSE system under different rounds.

In Table 2 and Figure 3, the study of the number of alive nodes (NOAN) of the ITSA-UCHSE method using current methods is given. The results implied that the ITSA-UCHSE technique reached increased values of NOAN under all rounds. For example, with 500 rounds, the ITSA-UCHSE algorithm reached a higher NOAN of 100 but the HHDAP, Q-DAEER and IPSO techniques resulted in a minimum NOAN of 95, 92 and 93, correspondingly. Additionally, with 600 rounds, the ITSA-UCHSE method acquired an increased NOAN of 100 while the HHDAP, Q-DAEER and IPSO methods had a reduced NOAN of 90, 74 and 91, respectively. Furthermore, with 700 rounds, the ITSA-UCHSE method reached a higher NOAN of 97 whereas the HHDAP, Q-DAEER and IPSO methods had a minimum NOAN of 63, 19 and 65, correspondingly.

**Figure 4:**
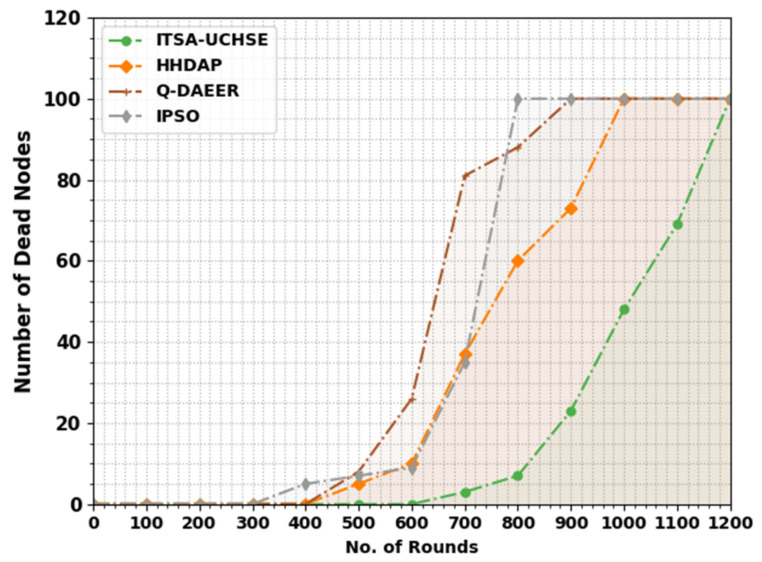
NODN analysis of ITSA-UCHSE system under distinct rounds.

Table 3 and Figure 4 exhibit the number of dead rounds (NODN) review of the ITSA-UCHSE method with existing techniques. The outcome exhibited that the ITSA-UCHSE algorithm has shown improved NODN values under every round. With 700 rounds, the ITSA-UCHSE technique attained a reduced NODN value of 3 while the HHDAP, Q-DAEER and IPSO techniques reached increased NODN values of 37, 81 and 35, correspondingly. Similarly, with 800 rounds, the ITSA-UCHSE algorithms gained a reduced NODN value of 7 while the HHDAP, Q-DAEER and IPSO methods reached increased NODN values of 60, 88 and 100, correspondingly.

**Figure 5:**
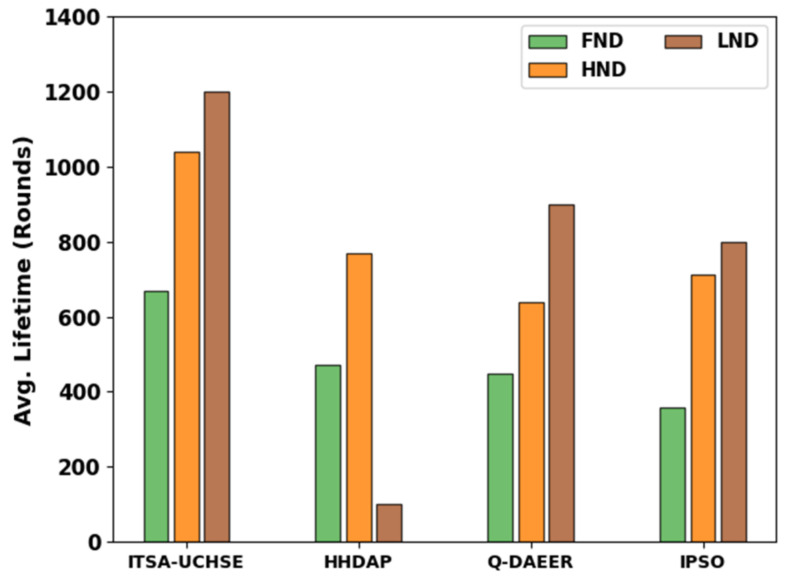
Average LFT analysis of ITSA-UCHSE system with other methods.

The average LFT assessment of the ITSA-UCHSE technique is demonstrated in Table 4 and Figure 5. Based on FND, the ITSA-UCHSE technique gained a higher FND of 668 rounds while the HHDAP, Q-DAEER and IPSO algorithms obtained a lower FND of 472, 448 and 359 rounds, respectively. Similarly, based on HND, the ITSA-UCHSE method attained a higher HND of 1041 rounds while the HHDAP, Q-DAEER and IPSO approaches obtained a lower HND of 768, 639 and 712 rounds, correspondingly. Furthermore, based on LND, the ITSA-UCHSE algorithm gained a higher LND of 1200 rounds while the HHDAP, Q-DAEER and IPSO methods gained a lower LND of 100, 900 and 800 rounds, respectively.

**Figure 6:**
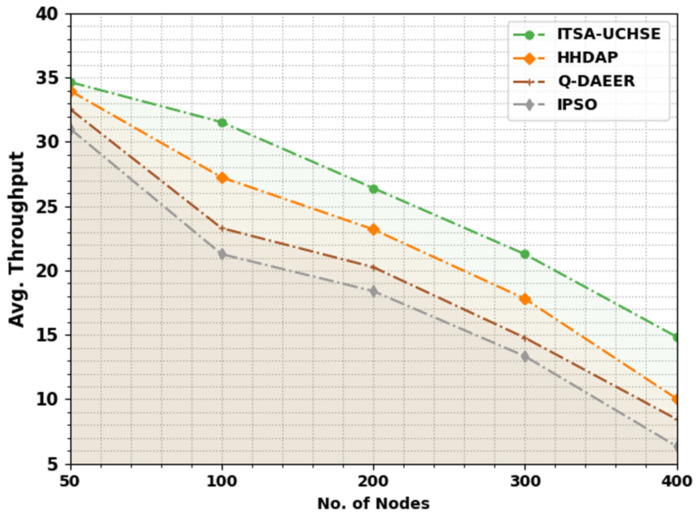
ATHRO analysis of ITSA-UCHSE system under distinct nodes.

In Table 5 and Figure 6, the average throughput (ATHRO) study of the ITSA-UCHSE method with recent models is given. The outcomes implied that the ITSA-UCHSE approach reached increased values of ATHRO in several nodes. For example, with 50 nodes, the ITSA-UCHSE method reached a higher ATHRO of 34.66 while the HHDAP, Q-DAEER and IPSO methods had a reduced ATHRO of 33.99, 32.56 and 31.04, correspondingly. Moreover, with 100 nodes, the ITSA-UCHSE method reached an increased ATHRO of 31.54, while the HHDAP, Q-DAEER and IPSO approaches resulted in a reduced ATHRO of 27.25, 23.29 and 21.27, correspondingly. Additionally, with 400 nodes, the ITSA-UCHSE technique reached an increased ATHRO of 14.86, while the HHDAP, Q-DAEER and IPSO methods resulted in a reduced ATHRO of 10.06, 8.46 and 6.36, correspondingly.

In Table 6, the total remaining energy (TRE) study of the ITSA-UCHSE method with recent methods is given. The outcomes exhibited that the ITSA-UCHSE technique reached increased values of TRE under all rounds. For example, with 100 rounds, the ITSA-UCHSE method reached a higher TRE of 98.14 while the HHDAP, Q-DAEER and IPSO techniques resulted in a reduced TRE of 97.09, 89.96 and 87.59, correspondingly. Furthermore, with 600 rounds, the ITSA-UCHSE approach had a higher TRE of 67.80, while the HHDAP, Q-DAEER and IPSO methods resulted in a reduced TRE of 59.89, 53.56 and 48.81, correspondingly. Additionally, with 1000 rounds, the ITSA-UCHSE method reached an increased TRE of 50.13, while the HHDAP, Q-DAEER and IPSO techniques resulted in a reduced TRE of 38.52, 19.26 and 17.15, correspondingly.

From the above-mentioned results and discussion, it is evident that the proposed model achieves reduced ECON values and increased lifetime, which assures one that the nodes with higher energy and lower distance can be chosen as CHs with proper cluster sizes. The effectual selection of cluster sizes balances the load among the clusters, which in turn increases reliability and reduces the risk of failure. In addition, the mitigation of hotspots can also reduce the cost of maintenance and repair. Moreover, the proposed model can help to extend the lifespan of the system and delay the need for replacement or upgrading. Therefore, the proposed model can be employed for accomplishing maximum energy efficiency and lifetime in the WSN.

## 5. Conclusions

This paper has developed a novel ITSA-UCHSE method for hotspot elimination in energy-aware WSNs. The ITSA-UCHSE technique is intended to resolve the hotspot problem and uneven energy dissipation in the WSN. Additionally, the ITSA is derived from the use of a tent chaotic map with the traditional TSA. In addition, the ITSA-UCHSE technique computes a fitness value based on energy and distance metrics. Moreover, the cluster size determination via the ITSA-UCHSE technique helps to address the hotspot issue. To demonstrate the enhanced performance of the ITSA-UCHSE methodology, a series of simulation analyses were conducted. The simulation values stated that the ITSA-UCHSE algorithm has acquired improved results over other models with maximum energy efficiency, increased network lifetime and enhanced throughput. As a part of future extension, the efficiency of the ITSA-UCHSE algorithm will be enriched via data aggregation techniques. In addition, the computation complexity of the proposed model can be examined in future.

## Figures and Tables

**Table 1:** ECON analysis of ITSA-UCHSE system with existing approaches under distinct nodes.

Energy Consumption (J)				
No. of Nodes	ITSA-UCHSE	HHDAP	Q-DAEER	IPSO
50	0.6666	0.7294	0.7811	0.8439
100	0.8402	0.9621	1.0545	1.1690
200	0.9621	1.1616	1.4387	1.5753
300	1.1173	1.3094	1.6936	1.8303
400	1.1690	1.3611	1.7379	1.9337

**Table 2:** NOAN analysis of ITSA-UCHSE system with current methods under different rounds.

Number of Alive Nodes				
No. of Rounds	ITSA-UCHSE	HHDAP	Q-DAEER	IPSO
0	100	100	100	100
100	100	100	100	100
200	100	100	100	100
300	100	100	100	100
400	100	100	100	95
500	100	95	92	93
600	100	90	74	91
700	97	63	19	65
800	93	40	12	0
900	77	27	0	0
1000	52	0	0	0
1100	31	0	0	0
1200	0	0	0	0

**Table 3:** NODN analysis of ITSA-UCHSE system with current methods under different rounds.

Number of Dead Nodes				
No. of Rounds	ITSA-UCHSE	HHDAP	Q-DAEER	IPSO
0	0	0	0	0
100	0	0	0	0
200	0	0	0	0
300	0	0	0	0
400	0	0	0	5
500	0	5	8	7
600	0	10	26	9
700	3	37	81	35
800	7	60	88	100
900	23	73	100	100
1000	48	100	100	100
1100	69	100	100	100
1200	100	100	100	100

**Table 4:** Average lifetime analysis of ITSA-UCHSE system with other approaches.

Avg. Lifetime in Rounds				
	ITSA-UCHSE	HHDAP	Q-DAEER	IPSO
FND	668	472	448	359
HND	1041	768	639	712
LND	1200	100	900	800

**Table 5:** ATHRO analysis of ITSA-UCHSE system with existing approaches under distinct nodes.

Avg. Throughput				
No. of Nodes	ITSA-UCHSE	HHDAP	Q-DAEER	IPSO
50	34.66	33.99	32.56	31.04
100	31.54	27.25	23.29	21.27
200	26.41	23.20	20.26	18.40
300	21.27	17.81	14.78	13.35
400	14.86	10.06	8.46	6.36

**Table 6:** TRE analysis of ITSA-UCHSE system with current methods under distinct rounds.

Total Remaining Energy (%)				
No. of Rounds	ITSA-UCHSE	HHDAP	Q-DAEER	IPSO
0	100.00	100.00	100.00	100.00
100	98.14	97.09	89.96	87.59
200	97.61	95.50	79.67	76.25
300	90.75	84.42	70.18	63.85
400	81.52	76.51	63.32	59.89
500	70.18	64.90	58.04	53.03
600	67.80	59.89	53.56	48.81
700	63.85	54.61	48.02	43.01
800	61.21	52.77	41.42	34.30
900	58.04	49.07	31.66	27.44
1000	50.13	38.52	19.26	17.15
1100	43.27	28.76	15.04	10.03
1200	37.20	26.39	13.46	6.34

## Data Availability

Data sharing does not apply to this article as no datasets were generated during the current study.
